# Study design of the Routine Outcome Monitoring for Geriatric Psychiatry & Science (ROM-GPS) project; a cohort study of older patients with affective disorders referred for specialised geriatric mental health care

**DOI:** 10.1186/s12888-019-2176-6

**Published:** 2019-06-17

**Authors:** Richard C. Oude Voshaar, Ton D. F. Dhondt, Mario Fluiter, Paul Naarding, Sanne Wassink, Maureen M. J. Smeets, Loeki P. R. M. Pelzers, Astrid Lugtenburg, Martine Veenstra, Radboud M. Marijnissen, Gert-Jan Hendriks, Lia A. Verlinde, Robert A. Schoevers, Rob H. S. van den Brink

**Affiliations:** 1 University of Groningen, University Medical Center Groningen, Groningen, The Netherlands; 2 Mental Health Center GGZ Noord-Holland Noord, Heerhugowaard, The Netherlands; 3 GGNet Mental Health, Division of Old Age Psychiatry, Warnsveld & Apeldoorn, The Netherlands; 4 Mental Health Center GGZ Centraal, Ermelo, The Netherlands; 5 Mental Health Center GGZ Drenthe, Assen, The Netherlands; 6 Mental health Center Pro Persona, Arnhem/Nijmegen, The Netherlands; 7 Mental Health Center Mediant, Enschede, the Netherlands

**Keywords:** Affective disorder, Depressive disorder, Anxiety disorder, Somatic symptom disorder, Aged, Aged, 80 years and over, Cohort, Treatment outcome, Routine outcome monitoring

## Abstract

**Background:**

Affective disorders, encompassing depressive-, anxiety-, and somatic symptom disorders, are the most prevalent mental disorders in later life. Treatment protocols and guidelines largely rely on evidence from RCTs conducted in younger age samples and ignore comorbidity between these disorders. Moreover, studies in geriatric psychiatry are often limited to the “younger old” and rarely include the most frail. Therefore, the effectiveness of treatment in routine clinical care for older patients and impact of ageing characteristics is largely unknown.

**Objective:**

The primary aim of the Routine Outcome Monitoring for Geriatric Psychiatry & Science (ROM-GPS) – project is to examine the impact of ageing characteristics on the effectiveness of treatment for affective disorders in specialised geriatric mental health care.

**Methods:**

ROM-GPS is a two-stage, multicentre project. In stage one, all patients aged ≥60 years referred to participating outpatient clinics for specialised geriatric mental health care will be routinely screened with a semi-structured psychiatric interview, the Mini International Neuropsychiatric Interview and self-report symptom severity scales assessing depression, generalized anxiety, hypochondria, and alcohol use. Patients with a unipolar depressive, anxiety or somatic symptom disorder will be asked informed consent to participate in a second (research) stage to be extensively phenotyped at baseline and closely monitored during their first year of treatment with remission at one-year follow-up as the primary outcome parameter. In addition to a large test battery of potential confounders, specific attention is paid to cognitive functioning (including computerized tests with the Cogstate test battery as well as paper and pencil tests) and physical functioning (including multimorbidity, polypharmacy, and different frailty indicators). The study is designed as an ongoing project, enabling minor adaptations once a year (change of instruments).

**Discussion:**

Although effectiveness studies using observational data can easily be biased, potential selection bias can be quantified and potentially corrected (e.g. by propensity scoring). Knowledge of age-related determinants of treatment effectiveness, may stimulate the development of new interventions. Moreover, studying late-life depressive, anxiety and somatic symptom disorders jointly enables data-driven studies for more optimal classification of these disorders in later life.

**Trial registration:**

Dutch Trial Register: NL6704 (www.trialregister.nl). Retrospectively registered on 2017-12-05.

## Background

Affective disorders, encompassing depressive, anxiety, and somatic-symptom disorders, are the most prevalent mental health disorders in later life. Reported prevalence rates centre around 10% for each of these groups of disorders, although studies vary due to differences in the age cut-off, time window, level of caseness, and diagnostic instrument applied [1–5]. The disease burden of affective disorders is high, as each of these disorders is associated with a lower quality of life, poorer prognosis of comorbid somatic diseases, and increased health care use [5–7]. Moreover, comorbidity between affective disorders is the rule rather than the exception, with up to 75% of the patients suffering from disorders out of at least two of the three diagnostic groups [8]. Comorbidity increases the disease burden more than can be explained by the sum of the individual disorders [9]. Despite these facts, comorbidity between affective disorders is largely neglected in disorder specific treatment guidelines as well as disorder specific care paths that have been implemented in mental health organisations.

Nowadays, the provision of mental health care averts 15% of the disease burden (measured as ‘years lived with disability’) caused by depressive disorders and 13% of the disease burden caused by anxiety disorders by 13% [10]. Over 50% of depressed patients across all age groups will not achieve remission with their first treatment [11]. Ultimately, 20–30% of depressed patients will not achieve full recovery despite access to multiple interventions [12]. Although affective disorders can be effectively treated in later life, data show that results with respect to depression as well as anxiety disorders worsen with increasing age [13–15]. Recovery may well be slower [16] and in case of remission, relapses more frequently occur compared to younger-aged patients [17]. Several explanations can be put forward why the effectiveness of treatment decreases with increasing age. Firstly, most treatment protocols have been developed in younger adults, ignoring specific age-related somatic comorbidities like somatic multimorbidity, physical frailty, and mild cognitive impairment, as well as age-related psychosocial adversities, like loss of a spouse or loneliness. Secondly, empirical data on the treatment of older persons with affective disorders, especially studies addressing specific characteristics of older persons, is hardly available. To our knowledge, no randomised controlled treatment trials have been conducted for somatization in later life [18] and only a few for late-life anxiety [19]. Most studies in late-life affective disorders have addressed the treatment of depression. Unfortunately, treatment response and remission are less likely in older compared to younger patients [20], and efficacy even decreases with age in samples of 60 years and older [13]. Findings that treatment efficacy of affective disorders decreases with the patients’ age, suggest that age-related characteristics may be involved. A systematic review, however, showed that geriatric syndromes (disability, multimorbidity, frailty, malnutrition) were hardly taken into account in studies testing the efficacy of antidepressants in later life [21]. Therefore, it remains difficult to what extent these characteristics may explain worse treatment results with increasing age as well as to what extent older patients included in treatment trials are representative for the patients we see in daily care. Considering that even in younger age groups, only 17–25% of the regular care patients would qualify for participation in antidepressant efficacy trials [22], this figure is probably much worse in later life.

Clinical cohort studies may fill the gap left by randomized controlled trials as they represent patients and outcomes in regular care rather than the optimized situation of an RCT. Cohort studies in psychiatry are, however, limited by convenience sampling, a focus on one type of disorder, restricted monitoring of treatment provided, and long intervals between assessments (e.g. [23, 24]). Moreover, in later life, cohort studies of anxiety disorders and somatoform disorders are lacking.

Routine outcome monitoring (ROM) which has been implemented in different services in the Netherlands and internationally offers another possibility to assess the effectiveness of protocolled interventions in daily care that were implemented on the basis of evidence from RCTs in selected populations [25]. Unfortunately, an optimal scientific study design may conflict with the practical use of ROM systems to facilitate routine clinical care. Whereas assessment of only one outcome measure may be sufficient for ROM application in routine clinical care [26], the answer to a broader range of (fundamental) research questions may be hindered by inadequate confounder control, selective participation and dropout, and low quality of diagnostic assessment [27, 28]. Incorporating all the necessary instruments in day-to-day ROM systems will increase patient burden and translates to higher number of missing data and early dropouts, again threatening the external validity of extended ROM data.

To overcome the limitations of traditional clinical cohort studies and ROM, we have designed the Routine Outcome Monitoring for Geriatric Psychiatry & Science (ROM-GPS) project. ROM-GPS is a two-stage project. The first stage is simply an improvement of routine clinical care by extending the diagnostic intake at specialised mental health outpatient clinics for older persons with a standardised diagnostic assessment performed by trained personnel independent of the health care professional who is responsible for intake and treatment. In case patients have an affective disorder, they are asked for informed consent to participate in the second stage of ROM-GPS. The second stage is a cohort study with an extended baseline assessment, follow-up assessments and treatment monitoring.

### Objectives

The primary aim of the ROM-GPS project is to study the one-year outcome (remission) of late-life depressive-, anxiety- and somatic symptom disorders jointly. ROM-GPS specifically aims to estimate the impact of comorbidity between affective disorders, the treatment components delivered, and age-related characteristics on treatment effectiveness. Such knowledge will guide the development of more effective and age-specific treatment strategies.

## Methods/design

### Study design

ROM-GPS offers an infrastructure for collecting high-quality research data on treatment effects for late-life affective disorders in specialised mental health care, with detailed assessment of the delivered treatment (see below). This infrastructure is established in the Northern and central regions of the Netherlands.

ROM-GPS is in fact a clinical cohort study, enriched with principles of ROM, designed as a two-stage project. Designated outpatient mental health clinics for geriatric psychiatry have standardized and protocolized their intake procedure for new referrals by adding a diagnostic assessment by a research nurse within two weeks after the initial contact with a mental health professional. The research nurse will administer the semi-structured psychiatric interview, based on the Mini International Neuropsychiatric Interview Plus (MINI-Plus, see below), for a full diagnostic assessment [29, 30], as well as the Montreal Cognitive Assessment (MoCA) [31], a brief cognitive screening tool. To guarantee high data quality, research nurses are well-trained at the Rob Giel Research center. Prior to formal data collection, research nurses have to attend a one-day training class as well as to observe two intakes of an experienced ROM-GPS research nurse. Moreover, the first two assessments will be supervised by a senior ROM-GPS research nurse to check procedures and data quality. During the study, research nurses participate in three obligatory training days each year, and receive regular supervision by a geriatric psychiatrist. In addition to the adapted MINI-Plus interview, four self-report questionnaires are administered to assess severity of depressive symptoms (30-item Inventory of Depressive Symptoms, IDS) [32], anxiety symptoms (Geriatric Anxiety Inventory, GAI) [33], hypochondria (Whitley Index, WI) [34, 35], and alcohol usage (Alcohol Usage Identification Test, AUDIT) [36]. These questionnaires have been chosen for their applicability and good psychometric properties in an older population.

Patients who meet the in- and exclusion criteria, will be informed orally and in writing about the research part of the project. After (at least) one week, patients will be asked whether they are interested to participate in the second stage of the project. For this stage informed consent is to be signed, where after a detailed assessment of patients is performed, including the administration of some extra observer-rated and self-report questionnaires, a brief physical examination, and a cognitive test battery (for overview see Table 1, for additional information see below). These patients will be monitored every four months by postal questionnaires (see also Table 1), which focus on re-assessment of psychiatric symptom severity. At one-year follow-up or end of treatment if treatment is concluded earlier, all baseline characteristics amenable to change will be reassessed during a site-visit. Two years after the detailed assessment at baseline, a postal questionnaire, similar to the four-month assessments will be conducted as a last follow-up assessment.

**Table 1:** Measurements of the ROM-GPS project

Characteristics	Instruments	Measurements points				
		Intake	Baseline	Repeated measures (per 4 months)	One-year follow-up (or end of treatment)	Two-year follow-up
Psychopathology						
• Psychiatric diagnoses	Mini International Neuropsychiatric Interview (MINI-Plus)	X			X	
• Depressive symptoms	Inventory of Depressive symptoms (IDS)	X		X	X	X
• General anxiety level	Geriatric Anxiety Inventory (GAI)	X		X	X	X
• Hypochondria	Whitley Index (WI)	X		X	X	X
• Worrying	Penn State Worry Questionnaire (PSWQ)		X	X	X	X
• Agoraphobic avoidance	Mobility Inventory (MI)		X	X	X	X
• Social anxiety	Leibowitz Social Anxiety Scale (LSAS)		X	X	X	X
Psychiatric treatment						
History of psychiatric treatment	Semi-structured interview		X			
Current psychiatric treatment	Checklist treatment modalities and intensity				X	
Psychosocial characteristics						
Socio-demographics	Age, sex, level of education, income, partner status		X		X	
BIG-five personality traits	NEO-Five Factor Inventory		X			
Recent life-events	Brugha Questionnaire		X		X	X
Early life-events	NEMESIS Questionnaire		X			
Social network size	Social Network Index		X		X	
Loneliness	De Jong-Gierveld scale		X		X	
Lifestyle						
Alcohol usage	Alcohol Use Disorder Identification Test (AUDIT)		X		X	X
Smoking	Past & current smoking questionnaire		X		X	
Sleep	Insomnia Rating Scale (IRS)		X		X	
Physical activity	Int. Physical Activity Questionnaire (IPAQ-short form)		X		X	
Self-management abilities	Self Management Abilities Scale (SMAS-30)		X		X	
Cognitive functioning						
Global cognitive functioning	Montreal Cognitive Assessment (MoCA) battery	X				
Cogstate battery	Several computerised tests (www.cogstate.com)		X		X	
Verbal memory	10 words test		X		X	
Processing speed & executive functioning	Stroop Coloured-Word test – short form		X		X	
Working memory	Digit span		X		X	
Physical functioning						
Chronic diseases	LASA questionnaire		X			
Functional limitations	WHO-Disability scale (WHO-DAS II)		X		X	X
Medication	Registration of (prescribed) drug usage		X		X	
Weight, length, waist circumference, blood pressure	Physical examination		X			
AGE accumulation in skin	AGE reader		X		X	
Biomedical frailty	Fried frailty index (gait speed, handgrip strength)		X		X	
Broad frailty phenotype	Tilburg Frailty Indicator		X		X	X
Routine blood chemistry	Hb, Ht, MCV, TC, LDL, HDL, TG, Kreat, Albumin, TSH, FT4		X			

ROM-GPS is designed as an ongoing project, allowing to replace or add specific measurements once a year. ROM-GPS has started on 1 January 2015 at the department of psychiatry of the University Medical Center Groningen and GGZ Noord-Holland Noord.

### Participants and setting

The domain population for the ROM-GPS project are all patients aged 60 years and older who are referred to one of the participating outpatient clinics for specialised geriatric mental health care, i.e. the outpatient clinic for geriatric psychiatry of the University Medical Center Groningen and affiliated mental health centers (GGZ Noord-Holland Noord per 01-01-2015, GGNet per 01-09-2016, GGZ Centraal per 01-01-2017, GGZ Drenthe per 01-04-2017, Mediant per 01-02-2018, and Pro Persona per 01-03-2019).

Inclusion criteria for the research part of the project (stage II) are 1) an age of 60 years or older, 2) the presence of an affective disorder confirmed by the Mini International Neuropsychiatric Interview-Plus (MINI-Plus), and 3) given informed consent.

As the MINI-Plus has been developed for the assessment of DSM-IV disorders, we have made some minor adaptations in the sections for mood and anxiety disorders to meet DSM-5 criteria for these disorders. Unipolar depressive disorders that will be assessed are: unipolar major depressive disorder (single /recurrent episode), persistent depressive disorder (dysthymia), and depressive disorder due to another medical condition. Anxiety disorders that will be assessed are panic disorder, agoraphobia, social anxiety disorder and generalized anxiety disorder. Bipolar disorders will be assessed (but will lead to exclusion of the patient, see below). Obsessive-compulsive disorder and posttraumatic stress disorder will also be assessed (but only allowed as comorbidity for inclusion in the study). As the DSM-IV section of somatoform disorders (DSM-IV) has been replaced by the DSM-5 section of somatic symptom disorders (DSM-5), we made major adaptations for this part of MINI-Plus especially new questions for assessing a somatic symptom disorder. Therefore, we will assess only the presence of a somatic symptom disorder, including the specifier with predominant pain, as well as illness anxiety disorder (similar to hypochondria in DSM-IV).

Finally, subthreshold depression and panic disorders will be considered (as comorbid disorders). Subthreshold depression will be defined according to the DSM-IV-TR research criteria for minor depression. Subthreshold panic disorder will be established by skipping the rules for panic disorders with respect to absence of a situational trigger of the panic attacks, peaking of the attacks within 10 min, and minimum number of somatic or cognitive symptoms required (1 instead of 4), enabling us to also report on atypical panic attacks and panic attacks with limited symptoms.

Exclusion criteria for the research part of the project (stage II) are 1) an established diagnosis of a neurodegenerative disorder or less than 18 points on the Montreal Cognitive Assessment (MoCA) test, 2) a (history of a) bipolar or psychotic disorder, 3) a severe substance-use disorder in need of specialised treatment, 4) physically or mentally too handicapped to administer self-report questionnaires or perform cognitive testing, or 5) insufficient mastery of the Dutch language. The MoCA is a short screening instrument for cognitive function [31], which covers the domains attention and concentration, memory, orientation, language, visuoconstructional skills, conceptual thinking, calculations and executive functions. Meta-analysis has shown that a cut-off of 23 out of 30 points differentiates best between healthy persons and those with a neurocognitive disorder [37]. As affective disorders, primarily depressive and generalized anxiety disorder, may interfere with cognitive functioning, we only excluded patients scoring less than 18 points (whose score is classified as indicating ‘moderate [score 10-17] or severe [less than 10] cognitive impairment’ versus ‘mild cognitive impairment [18–26] or normal [more than 26]; although validation of these severity ranges in a psychiatric sample has not been established yet; see http://www.mocatest.org/faq/). At the start of the project, we originally included the Mini Mental State Examination (MMSE) [38], but replaced the MMSE by the MoCA per 01-01-2016, as the MoCA explicitly covers executive functions in contrast to the MMSE, which is associated with late-onset depressive disorder and late-life anxiety disorder and therefore particularly relevant to reassess at one year follow-up.

### Outcome variables

- The primary outcome variable is remission of the index affective disorder (yes/no), assessed with the MINI-Plus at one-year follow-up or end of treatment if specialized mental health care is concluded earlier.
- Secondary outcome measures include the course of symptom severity of the index disorder which will be measured every four months by well-validated self-report questionnaires (see Table 1).
  Depressive symptom severity will be monitored with the 30-item self-rating Inventory of Depressive Symptomatology (IDS), which has adequate psychometric properties [32]. The sum score ranges from 0 to 60; the severity of depression can be classified as none (score range 0 through 12), mild (13 though 24), moderate (25 through 37), severe (38 through 47) and very severe (48 or higher). Severity of somatic symptom disorders is assessed with the Whitley Index (WI) [39]. The WI has 14 statements (yes/no) addressing the severity of hypochondriacal cognitions and somatization symptoms [39]. The course of anxiety disorders will be monitored with the 20-item Geriatric Anxiety Inventory, which has specifically been developed for measuring anxiety in older persons [33]. In addition, disorder specific instruments will be applied to monitor the severity of the individual anxiety disorders. The Mobility Inventory – Avoidance scale (MI-A) [40] will be administered to monitor agoraphobic severity in patients with panic disorder or agoraphobia, the Penn State Worry Questionnaire (PSWQ) [41] to monitor the course of generalized anxiety disorder, and the Leibowitz Social Anxiety Scale (LSAS) [42] for the course of social anxiety.
- Functional limitations will be assessed at baseline and one year follow-up with the WHO-Disability scale (WHO-DAS II) [43].
- As all secondary outcome variables are self-report measures, these will be re-administered at two-year follow-up by a postal questionnaire.

### Primary determinants

As determinants of treatment effectiveness, we will examine the different treatment modalities and their intensity offered to patients as well as age-related physical and cognitive patient characteristics.

- Psychiatric treatment is assessed at baseline and one-year follow-up with the aid of a self-developed checklist of treatment modalities and intensity. We will administer the number of contacts per discipline (physician, psychiatrist, psychologist, etc.), setting (outpatient clinic, home visits, day care, inpatient care), and content of treatment provided. The content will be classified as drug treatment, psychotherapeutic treatment or structured/supportive treatment. Structured/supportive treatment is defined as all non-pharmacological treatment delivered by health care professionals with a degree below a Master of Science/Arts level (the minimum level for psychologists and physicians). In case of drug treatment, we will collect the generic name, Anatomical Therapeutic Chemical (ATC) code, dose, and whether blood levels were assessed. In case of psychotherapeutic treatment, the psychotherapy will be classified as cognitive behavioral therapy, interpersonal psychotherapy, problem solving treatment, mindfulness based cognitive therapy, or other. At one-year follow-up, the research nurse will collect these data from the (electronic) medical records for each of the 4-month follow-up periods. Since reimbursement of mental health care in the Netherlands is based on the number of minutes spent by each mental health professional, registration of these data is considered accurate.
- Multimorbidity will be measured by inquiring about the most common chronic somatic diseases, using a validated self-report questionnaire previously used in the Netherlands Study of Depression in Older persons (NESDO) and validated in the Longitudinal Aging Study Amsterdam (LASA) [23, 44]. Prescribed drug use at baseline and one-year follow-up will be registered. For each drug taken in the past month, we will assess the generic name, ATC-code, frequency of use, daily dosage, and in case of psychotropic drugs, also duration of use. This enables to examine the number of prescribed drugs (or specific polypharmacy definitions) as determinant of treatment outcome. For more specific research questions, the number of prescribed drugs, changes over one year or specific (somatic) drugs, can be included as covariate.
- Frailty will be assessed according to both a biomedical and a broad phenotype at baseline and one-year follow-up. The physical frailty phenotype will be assessed according to Fried [45]. This Fried Frailty Index consists of five criteria and physical frailty is considered present when three or more criteria are met, i.e. 1) unintentional weight loss of more than 4.5 kg in the past year, 2) low level of physical activities assessed with the International Physical Activity Questionnaire (IPAQ, see below), 3) exhaustion, based on a positive answer on one out of two items of the Center for Epidemiological Studies Depression scale, 4) low grip strength according to body mass index and sex, assessed with a dynamometer and 5) low gait speed according to body length and sex.
  Secondly, the Tilburg Frailty Indicator (TFI) will be administered to assess the broad frailty phenotype [46, 47]. The TFI is a self-report questionnaire, based on a Delphi study among a panel of international frailty experts and validated in cross-sectional studies.
  In addition, a physical examination will be performed to assess weight (kg), length (cm), waist circumference (cm), blood pressure (mmHg), the metabolic syndrome according to the National Cholesterol Education Program (NCEP)-Adult Treatment Panel III guidelines [48] and the accumulation of advanced glycation endproducts (AGE) in the skin reflecting lifetime metabolic dysregulation [49, 50].
- In addition to the MoCA, cognitive functioning will be more extensively tested during baseline and one-year follow-up. Firstly, we will apply the computerized Cogstate test battery (http://www.cogstate.com), including the Detection Test measuring psychomotor function, the Identification Test measuring attention, the One Card Learning Test measuring visual learning, and the One Back Test measuring working memory. In addition to these computerized tasks, we also administer some traditional paper and pencil tests [51]. These include an abbreviated version of the Stroop Colour-word test [52] for processing speed and executive functioning, the subtest Digit Span from the Wechsler Adult Intelligence Scale [53] for working memory and a modified version (10-words test) of the Auditory Verbal Learning Test [54, 55] for verbal memory.

### Secondary determinants and potential confounders

As potentially interesting determinants and confounding variables, we will assess age, sex, level of education, partner status, the BIG-five personality traits, early and recent life-events, extent of social network, loneliness, lifestyle characteristics (including alcohol usage, smoking, sleep, physical activity) and self-management abilities.

The Big Five personality traits –openness to experience, conscientiousness, extraversion, agreeableness and neuroticism- will be assessed using the NEO Five Factor Inventory (NEO-FFI) [56]. Negative life events in childhood and adolescence concerning emotional, physical and sexual abuse were will measured with the NEMESIS questionnaire [57]. The list of threatening experiences [58] will be used to inquire about 12 categories of negative life events in the past year, such as the death of a family member, divorce or financial problems.

Extent of social network and loneliness will be assessed with the Social Network Index [59] and the 11 item Loneliness scale [60], respectively.

Assessment of lifestyle characteristics will include smoking, alcohol use, sleep, and physical activities. Smoking behaviour is assessed with standard questions, and the use of alcohol is assessed using the Alcohol Use Disorders Identification Test (AUDIT) [36]. Sleep will be assessed with the Insomnia Rating Scale (IRS) [61]. Finally, the International Physical Activities Questionnaire (IPAQ) [62] will be used to measure energy expenditure based on sports and daily activities. Abilities for self-management of wellbeing will be measured with the Self-Management Abilities Scale (SMAS-30) [63].

Table 1 presents an overview of all instruments applied, including the moment of administration, i.e. at intake (stage one), at baseline (start of stage two), every four months during the first year of treatment, at one-year follow-up or end of treatment when treatment is concluded within one year, and finally at two-year follow-up.

### Statistical analyses

The effect of a determinant on the primary outcome, remission of the index disorder at one year follow-up, will be examined by logistic regression analysis. Depending on the specific objective of specific studies that will be embedded in ROM-GPS, comorbidity between affective disorders can be addressed in different ways. Firstly, comorbidity can be included as a covariate to adjust for the presence of comorbidity. Secondly, by having a sample of well-phenotype patients, a sensitivity analysis on patients with a ‘pure’ affective disorder can be performed. Finally, if theoretically grounded, our research design also allows to examine the interaction between specific comorbid affective disorders and other determinants.

The effect on the secondary outcome, course of symptom severity, will be examined by random coefficient analysis, a specific type of linear mixed models, which takes into account that the repeated assessments of symptom severity are nested within subjects [64]. In random coefficient analysis, the development in the outcome variable is estimated by a straight line and the effect of the determinant on this linear development is tested by the interaction of the determinant with the time of assessment of the outcome variable. All analyses will be controlled for potential confounders.

### Power calculation

Applying logistic regression analysis for the primary outcome variable remission at one-year follow-up, requires a sample size of 231 patients to demonstrate the effect of a continuous determinant with an odd ratio of 1.4 per standard deviation (which is equivalent to a small effect size d of 0.2) with a power of 80% and a significance level of 5%, assuming a one-year remission rate of 50% at the mean of the determinant [65, 66]. With a sample size of 231 patients, ordinary linear regression can demonstrate the effect of a continuous determinant which explains 4% of variance in change in a continuous outcome variable (e.g. change between baseline and follow-up on the secondary outcome measures) with a power of 87%. This power further increases when multiple repeated assessments of the outcome variable (e.g. the four-monthly assessments of symptom severity) are examined as outcome using random coefficient analysis.

To achieve a sample of 231 patients with a complete baseline and follow-up assessment, we estimate that 289 patients have to give informed consent assuming a 20% dropout rate at one-year follow-up [67]. Moreover, assuming an overlap between depressive, anxiety and somatic symptom disorders of at least 50%, analyses per diagnostic main group can be performed when approximately 600 patients have completed the study protocol.

### Ethical issues

The first stage is implemented at the participating outpatient clinics as part of routine clinical care. All patients of the participating mental health organisations are informed in writing at intake that data collected as part of routine clinical care may anonymously be used for research. When the first stage shows that patients meet the in- and exclusion criteria of the study, they are informed orally and in writing about the second stage of the project and are asked whether they may be contacted after one week, to hear whether they want to participate in the study. If so, an appointment is made for the baseline assessment, which starts with and only continues when written informed consent for the study is provided.

Before the start, all relevant documents have been submitted to the ethical review board of the University Medical Center Groningen (METc 2014/106), which concluded that the project is fully in line with the Dutch law (NL47717.042.14). ROM-GPS has been registered at the Dutch Trial Register (NTR6874) (www.trialregister.nl).

### Data-management

Data collection is supported by an online data entry and management system, i.e. Routine Outcome Quality Assessment (ROQUA, see https://www.roqua.nl), developed by the University Medical Center Groningen and the Rob Giel Research center. In case of a self-report questionnaire, patients can choose whether they want to fill in the questionnaire on the computer or prefer a paper and pencil version (which is entered in the online system afterwards by the research nurse).

## Discussion

### Relevance of expected findings

ROM-GPS will produce important new information about the actual effectiveness of interventions for late-life affective disorders in day-to-day mental health care. The population aged 60 or over is growing faster than all younger age groups. Globally, the number of persons aged 60 years will more than double by 2050 to 2.1 billion [68] of which at least every tenth will be confronted with common mental disorders [1–5] and may seek treatment. Knowledge regarding real life outcomes of those treatment is highly relevant in light of the disease burden of this group of disorders. Current treatment strategies, even under optimal circumstances, only achieve modest effects. In depth knowledge of ageing-related determinants as well as their relative contribution on treatment outcome and negative health consequences, may facilitate adaptation of current treatment strategies to address the most important determinants or their consequence more targeted and thus guide the development of new interventions for specific subgroups [69, 70]. Promising examples include psychotherapy adapted to (mild) cognitive deficits [71], optimizing cardiovascular treatment [72], or addressing biomedical frailty [69, 73]. In case of biomedical frailty, for example, antidepressants should be avoided to prevent polypharmacy and falls, while psychological treatment should also address coping with an ageing body (e.g. physiological exhaustion) and should preferably augmented with components of geriatric rehabilitation like protein rich diets and physical exercise. Results may thus directly guide clinical care and may lead to improvement of guidelines by early detection of treatment resistant groups.

ROM-GPS is worldwide the first study in which late-life depressive, anxiety and somatic symptom disorders are studied jointly using a unique hybrid design that combines the possibilities of ROM with more rigorous standardized assessments and follow-up measures. ROM-GPS adds to current practice as the use of (semi-)structured diagnostic psychiatric interviews is not standard practice in the Netherlands. Moreover, only a minority of patients receive ROM [27], despite recommendations in care standards and guidelines [www.ggzstandaarden.nl]. Furthermore, in case ROM is systematically implemented, only a few outcome instruments are used (comparably to our 4 and 8 month follow-up) [74], but no extensive baseline and outcome assessment as implemented in ROM-GPS for those patients giving informed consent. Therefore, our data collection enables to examine the impact of comorbidity on treatment outcome and disease burden. Hopefully, data collection may also assist the development of a better classification for affective disorders in later life. DSM- and ICD-classification systems are often criticized for the (potential) lack of age-neutrality of the criteria for mental disorders [75, 76]. Empirical studies supporting a more optimal age-specific classification, however, are hardly available. Since ROM-GPS includes three groups of extensively phenotyped affective disorders, application of data-driven techniques may be employed to support the development of a classification system that fits more accurately the affective symptoms experienced by older persons.

### Methodological considerations

By collecting these data with the aid of well-trained nurse practitioners or psychologists, the quality of the psychiatric diagnosis of both participants and non-responders is high. Since the first diagnostic assessment is done within the framework of care, research will feel like less of a burden to patients. Moreover, clinicians and patients will benefit as diagnostic data will become directly available for the mental health team. During stage one, data of all patients referred to the participating outpatient clinics are gathered. These data enable us to examine the representativeness of our study sample. In case of selection bias due to differential refusal rates between specific subgroups, findings may be adjusted using ‘propensity score matching’ [77]. To do so reliably, still sufficient numbers of participants are necessary of such subgroups.

The main limitation is probably the burden of the baseline assessment for patients giving informed consent (stage 2). This assessment takes approximately 3–4 h, which may affect patients’ willingness to participate. Nonetheless, our experience with the NESDO study reveals that many patients actually appreciate the thorough investigations done as part of clinical research [23]. Moreover, the assessment can be spread over 2 visits if necessary.

Secondly, we aim to conduct the extensive baseline assessment as soon as possible, but for pragmatic reasons, we consider a period up to 14 days as acceptable. Since symptoms my readily change after the first contact with specialised mental health care, this delay might confound results. Nonetheless, as the time between intake and baseline assessment is known, we can adjust for this time period and where relevant, also can perform sensitivity analyses on the subgroup that was assessed within one week.

### Final conclusion

The ROM-GPS study will show how effective the treatments are which are routinely provided in outpatient geriatric psychiatry, and to what extent this effectiveness is compromised by the typical characteristics of an elderly population. This will indicate whether standard treatments need to be adjusted and supplemented. Furthermore, it will provide a good infrastructure for patient selection for additional studies in our Regional Geriatric Psychiatry Network.

## Data Availability

Researchers interested in using the data, can contact the principle investigator. Data will only be made available in collaboration with the principle investigator and only in case a good research question is formulated, including a hypothesis and an elaborated statistical plan.

## Abbreviations

AGE: Advanced glycation endproducts
ATC code: Anatomical Therapeutic Chemical code
AUDIT: Alcohol Use Disorder Identification Test
DSM: Diagnostic and Statistical Manual of Mental Disorders
GAI: Geriatric Anxiety Inventory
ICD: International classification of diseases
IDS: Inventory of Depressive Symptoms
IPAQ: International Physical Activity Questionnaire
LASA: Longitudinal Aging Study Amsterdam
LSAS: Leibowitz Social Anxiety Scale
MI-A: Mobility Inventory – Avoidance scale
MINI-plus: Mini international neuropsychiatric interview, plus version
MoCA: Montreal Cognitive Assessment
NCEP: National cholesterol education program
NEMESIS: Netherlands mental health survey and incidence study
NEO-FFI: NEO Five Factor Inventory
NESDO: Netherlands study of depression in older persons
PSWQ: Penn State Worry Questionnaire
RCT: Randomised controlled trial
ROM: Routine Outcome Monitoring
ROM-GPS: Routine Outcome Monitoring for Geriatric Psychiatry & Science
SMAS-30: Self-management abilities scale – 30 item version
TFI: Tilburgse frailty index
WHO-DAS: World Health Organisation – Disability Assessment Schedule
WI: Whitley index
